# Sleep-Energy: An Energy Optimization Method to Sleep Stage Scoring

**DOI:** 10.1109/ACCESS.2023.3263477

**Published:** 2023-03-31

**Authors:** Bruno Aristimunha, Alexandre Janoni Bayerlein, M. Jorge Cardoso, Walter Hugo Lopez Pinaya, Raphael Yokoingawa De Camargo

**Affiliations:** 1 Center for Mathematics, Computing, and Cognition (CMCC), Federal University of ABC (UFABC), São Paulo 09210-580, Brazil; 2 Department of Biomedical Engineering, School of Biomedical Engineering and Imaging Sciences, King’s College London, WC2R 2LS London, U.K.

**Keywords:** Automated sleep staging, energy optimization, deep learning, electroencephalogram

## Abstract

Sleep is essential for physical and mental health. Polysomnography (PSG) procedures are labour-intensive and time-consuming, making diagnosing sleep disorders difficult. Automatic sleep staging using Machine Learning (ML) - based methods has been studied extensively, but frequently provides noisier predictions incompatible with typical manually annotated hypnograms. We propose an energy optimization method to improve the quality of hypnograms generated by automatic sleep staging procedures. The method evaluates the system’s total energy based on conditional probabilities for each epoch’s stage and employs an energy minimisation procedure. It can be used as a meta-optimisation layer over the sleep stage sequences generated by any classifier that generates prediction probabilities. The method improved the accuracy of state-of-the-art Deep Learning models in the Sleep EDFx dataset by 4.0% and in the DRM-SUB dataset by 2.8%.

## I Introduction

Sleep is one of the fundamental cognitive tasks performed by the brain for keeping physical and mental health [1]. Sleep disorders can be associated with multiple health problems, including psychiatric, immune, cardiovascular, metabolic, and sexual dysfunctions [1], [2], [3], [4], [5], [6], [7], [8]. The standard gold test for diagnostic sleep disorders is the polysomnography (PSG), where the subject spends a whole night with several electrodes and sensors attached to the body (*i.e*., Electroencephalogram - EEG, Electrooculogram - EOG, Electromyography - EMG, Electrocardiogram - ECGs, airflow, and blood oxygenation) [9]. A well-trained technician or physician then uses the captured time series to categorise each 30-second epoch in one of the multiple sleep stages, in a process called sleep scoring or sleep staging [10]. This labelling process is labour-intensive, time-consuming, and subject to errors and variability. This laborious process is a significant bottleneck that prevents more widespread testing in the population, resulting in the underdiagnosis of several sleep conditions.

Given the characteristic brain patterns governing the sleep stages, automatic sleep staging based on EEG has been studied extensively [9], [11], [12]. The first step is pre-processing the PSG signals, applying normalization, detrending, and band-pass filters, and performing artefact removal [13]. The next step is feature extraction, which generates a set of temporal, spectral, time-frequency, spatial, and other features. Finally, one can use different machine learning (ML)-based methods over the extracted features, such as decision trees [14], Hidden Markov Models [15], Support Vector Machines [16], Self-Organizing Maps [17], Random Forests [18], and more recently, Deep Learning Models [19], [20].

Deep learning models learn to identify important features in the EEG signals and use these features to classify the sleep stages when provided with sufficient training examples [19], [20]. The recent increase in available public datasets [21] enabled these methods to achieve state-of-the-art performance on sleep staging [22].

Adoption in clinical practice requires automatic sleep staging methods to have similar reliability levels to trained technicians and physicians. Most state-of-the-art models classify each 30-second epoch in isolation or considering only the immediate neighbouring epochs. However, in clinical practice, a broader context regarding the sleep stages around each epoch is also important and should be considered by models. For instance, Yang et al. [23] propose the use of a Hidden Markov Model (HMM) to improve the predictions made by a single electrode convolutional neural network by exploring sleep stage transition probabilities.

In this work, we propose an energy optimisation method called sleep-energy to improve the quality of hypnograms generated by automatic sleep staging procedures. The method evaluates the system’s total energy based on conditional probabilities for each epoch’s stage and employs an energy minimisation procedure. The energy comprises conditional probabilities from the ML model stage predictions, the prevalence of each sleep stage, and the stage transition probabilities. One can apply this method as a meta-optimisation layer over the sleep stage sequences generated by any ML classifier that outputs class probabilities, including state-of-the-art Deep Learning models. The highlights of our study are:

1) Our method can be used as a meta-optimisation layer to improve sleep stage sequence predictions from any ML-based model that generates prediction probabilities;
2) Our energy optimisation method reduces incoherent sleep stage transitions and improves the distribution of less frequent stages, such as N2, N3, and REM stages, while using low computational resources;
3) The proposed method outperforms the postoptimisation based on Hidden Markov Model - HMM, under a subject-independent evaluation paradigm, using Sleep-EDFx [24] and DRM-SUB [25] datasets.
4) To the best of our knowledge, this is the first use of an energy optimisation method on a sleep-scoring task or other EEG-based classification tasks.

## II Proposed Method

### A Automatic Sleep Staging

Sleep is composed of multiple cycles of sleep stages, which repeat during the night: Wake (W), Rapid Eye Movements (REM, denoted as R in this study), and Non-REM Stages 1 (N1), 2 (N2) and 3 (N3).^1^ The final hypnogram should contain the sleep stage *s_t_* at each 30s epoch *t*, where *t* ∈ {1, 2, …, *T*}.

In typical setups, an ML model receives the EEG signal from an epoch *t* and returns the probabilities of these epochs belonging to each of the five possible sleep stages. When applied to the whole hypnogram, it generates a *predicted probability matrix* with shape (5, *T*). With this predicted probability matrix, we apply a *argmax* function to extract the most likely sleep stage according to the model, generating a candidate hypnogram. We denote the predicted probability matrix as *P_pred_* (*s_t_*) and use the notation $s_{t}^{pred}$ to indicate the stage with the highest probability.

One can also extract other information from the predictions, such as the *confusion matrix* containing the probabilities of mispredictions. This matrix contains the actual sleep stage in the rows and the predicted state in the columns. By normalising the sum of values on each column, each matrix entry will contain the probability of the actual stage being *s_t_* (row) given that the ML model predicted a state $s_{t}^{pred}$ (column). We represent it as $P_{conf}\left(s_{t}|s_{t}^{pred}\right)$.

During sleep, stage transitions have different probabilities. We construct a *transition probability matrix* by (i) measuring the frequencies of transitions among different stages; (ii) organising the source stages in the rows and the target stages in the columns; and (iii) normalising the sum of each row in the matrix. We use the notation *P_trans_*(*s*_*t*−1_ → *s_t_*) to denote the probability of transition from state *s* at time *t*−1 to state *s* at time *t*.

### B Sleep-Energy Optimisation Method For Sleep Staging

**Figure 1:**
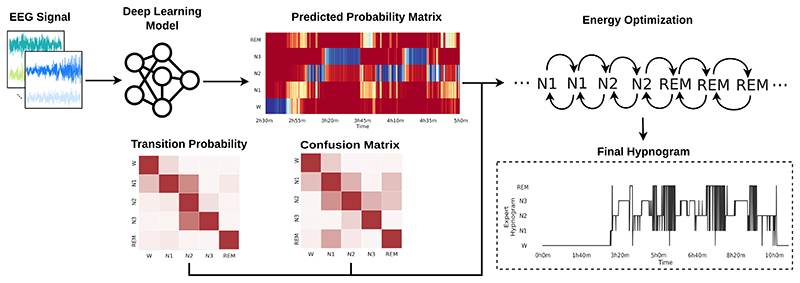
The general pipeline for the energy optimisation method. The ML model receives a sequence of 30s epochs and generates the predicted probability matrix. The energy optimiser uses this matrix to generate a candidate hypnogram and evaluates the system’s energy using the confusion and transition probability matrices. It then iteratively optimises the sleep stage sequence to reduce this overall energy and generates the final hypnogram.

In this work, we propose sleep-energy, an energy optimisation method that uses the prediction probability, confusion, and transition probabilities matrices to optimise the energy of the generated hypnogram. The method evaluates the initial energy of the system and iteratively optimises the sleep stage sequence to reduce this overall energy, as shown in Figure 1.

Although one typically selects the stage with the highest probability, there can be multiple stages with similar probabilities, or the predictions can be wrong. We leverage the information from transition probabilities, predicted probabilities, and confusion matrices to improve the quality of the constructed hypnogram. These matrices will indicate the likelihood for each stage *s* on each epoch *t*, based on different criteria: (i) the sleep stage at the previous epoch *t* −1, (ii) the confidence of the ML model prediction, and (iii) the errors of the ML model. For instance, we could have *P_pred_* (*s*, *t*) = 0.8, $P_{conf}\left(s|s_{t}^{pred}\right)=0.5$, *P_trans_*(*s*′ → *s*, *t*) = 0.1, and *P_trans_*(*s* → *s*′′, *t*+1) = 0.7. We would have a different set of probabilities for each sleep stage *s* ∈ {*W*, *N*1, *N*2, *N*3, *R*}.

We define the whole hypnogram as the variable to be optimised. To optimise the sequence of sleep stages *s_t_*, where *t* ∈ {1, 2, …, *T*}, we define an energy function *E*(*s_t_*) for each epoch. We consider the total energy of the hypnogram as the sum of *E*(*s_t_*) for all *t* and use an optimisation technique to minimise its value. We define the energy function as:

(1) $$\begin{array}{ll} E\left(s_{t}\right) & =\alpha *\left[e_{trans}\left(s_{t-1}\to s_{t}\right)+e_{trans}\left(s_{t}\to s_{t+1}\right)\right]\\ & +(1-\alpha )*\left[e_{conf}\left(s_{t}|s_{t}^{pred\;}\right)+e_{pred\;}\left(s_{t}\right)\right] \end{array}$$

where *α* ∈ [0, 1] indicates the relative contribution of the transition probability matrix in the energy function compared to the confusion matrix and prediction probabilities. The errors are given by:

(2) $$e_{trans\;}\left(s_{t-1}\to s_{t}\right)=\frac{1-\epsilon_{t}}{P_{trans\;}\left(s_{t-1}\to s_{t}\right)-\epsilon_{t}}-1$$

(3) $$e_{conf\;}\left(s_{t}|s_{t}^{pred\;}\right)=\frac{1-\epsilon_{p}}{P_{conf\;}\left(s_{t}|s_{t}^{pred\;}\right)-\epsilon_{p}}-1$$

(4) $$e_{pred\;}\left(s_{t}\right)=\frac{1-\epsilon_{p}}{P_{pred\;}\left(s_{t}\right)-\epsilon_{p}}-1$$

These equations have the format 1/*p*−1 so that the energy is zero when the respective probability *p* is one and increases when *p* goes to zero. To prevent divergence at *p* → 0 and limit its value, we include the constants *ϵ_t_* and *ϵ_p_*. When *p* = 0, the error will be 1/*ϵ_t_* − 1 and 1/*ϵ_p_* − 1, respectively. These constants also define the shape of the probability curve, with steeper curves for smaller *ϵ* values.

Our next step is to employ an optimisation technique for discrete search spaces to reduce the system’s energy. We employ a stochastic optimisation process to prevent the system from being stuck in local minima.

### C Energy Optimisation Using Simulated Annealing

We use an optimization strategy often used to model physical systems called *simulated annealing*. This strategy simulates the controlled heating and cooling of materials to change their properties in metallurgy applications. Translating into an optimization algorithm, the idea is to start the process with a higher temperature, initially permitting sleep stage transitions to higher energy (lower probability) stages and gradually reducing the temperature so that transitions to lower probability stages become less likely.

The process is conducted over multiple steps *k*, and the global state of the system at each moment is given by $S^{k}=\left(s_{1}^{k},s_{2}^{k},\ldots ,s_{T}^{k}\right)$. At each step of the simulated annealing process, the algorithm selects one candidate epoch *t* to update its value $s_{t}^{k}$. We update a single position at each step because it also changes the energy of its neighbours *t* − 1 and *t* + 1. The total energy of the hypnogram at update step *k* is:

(5) $$E\left(S^{k}\right)=\sum_{t=1}^{T}E\left(s_{t}^{k};S^{k}\right)$$

where $E\left(s_{t}^{k},t;S^{k}\right)$ is the energy for stage *s_t_* at the optimisation step *k* (Equation 1) when considering that the hypnogram is at global state *S^k^*.

We select the position *t* to update at each step *k* using the Boltzmann distribution, which depends on the state energy of each position $E\left(s_{t}^{k}\right)$ and the current temperature 1/*β_k_*. This distribution is the same as the softmax function and is given by:

(6) $$P_{update\;}(t)=1/Z*e^{-\beta_{k}*E\left(s_{t}^{k};S^{k}\right)}$$

where Z is the normalization factor, also called partition function, given by:

$$Z=\sum_{s^{\prime }=1}^{T}e^{-\beta *E\left(s_{s^{\prime }}^{k};S^{k}\right)}$$

Finally, the algorithm selects the new state $s_{t}^{k+1}$ for the selected position *t* using the same probability distribution:

(7) $$P\left(s_{t}^{k+1}\right)=1/Z*e^{-\beta *\text{Δ}E\left(s_{t}^{k+1};S^{k}\right)}$$

where *s*^*k*+1^ is one the 5 possible states {*W*, 1, 2, 3, *R*}, Z is the normalisation factor over these states, and $\text{Δ}E\left(s_{t}^{k+1};S^{k}\right)$ is the difference in the energy *E*(*S^k^*) of the hypnogram caused by the transition from state $s_{t}^{k}$ to the new state $s_{t}^{k+1}$:

(8) $$\text{Δ}E\left(s_{t}^{k+1};S^{k}\right)=\sum_{t^{\prime }=t-1}^{t+1}E\left(s_{t^{\prime }}^{k+1};S^{k+1}\right)-E\left(s_{t^{\prime }}^{k};S^{k}\right)$$

where *S*^*k*+1^ denotes the global state of the system at step *k* + 1 if the transition $s_{t}^{k}\to s_{t}^{k+1}$ occurs. We repeat the optimization process for *K* update steps, reducing the temperature 1/*β_k_* using a given annealing schedule.

## III Experimental Methodology

### A Datasets

We evaluated the energy optimisation method using two classical polysomnography datasets, the sleep EDFx expanded dataset, sleep cassette subset,^2^ and DREAMS subjects database (DRM-SUB) [25].^3^ The polysomnography exams contain two or three EEG electrodes (Fpz-Ca and Pz-Oz in the first dataset; FP1-A1, O1-A1, and Cz-A1 or C3-A1 depending of the subject in the second dataset), 1 EOG electrode, and 1 EMG electrode. We used only the EEG channels to perform the classification task. The EDFx dataset has 78 healthy subjects with ages between 25 and 101 and up to two sessions per subject, totalling 153 records [21], [24]. The DRM-SUB dataset contains 20 subjects with one whole night of polysomnography recording for each.

One specialist determined the corresponding sleep stage for each 30-second epoch in both datasets. Sleep stages were originally defined following the Rechtschaffen & Kales protocol and later converted to the AASM protocol, which has stages Wake, N1, N2, N3 and REM, which we denote {*W*, *R*, 1, 2, 3}. We re-sampled the EEG datasets to 100Hz. EEG series were normalised to a scale between ±1*μV* and combined with a temporal bandpass filter [0, 30] Hz to remove high-frequency noise and artefacts. We selected only the EEG channels to perform the classification task and discarded EOG and EMG data.

### B Model Training and Evaluation

We used the state-of-the-art Chambon [19] and U-Sleep [20] neural networks for sleep stage classification. Chambon is a small and efficient network with 11 layers, ten for feature extraction and one for classification. U-Sleep consists of an adapted U-Net for high-frequency features [26], with 11 encoding and 11 decoding layers. Both architectures delivered solid results in several machine learning paradigms and datasets [27], [28], [29], [30], [31].

We trained both neural network models from scratch with Xavier weight initialisation [32] and AdamW optimiser [33], with *β*_1_ = 0.9, *β*_2_ = 0.999, learning rate 1^−3^, weight decay 0.0005 (following [33] recommendation), and Cross-Entropy Loss. We used batch size 32 and 300 epochs, with early stopping regularisation and 30 epochs patience, following the methodology proposed by [31] for systematic comparisons.

We used subject-wise 5-fold cross-validation, with 20% of the subjects for testing, and split the remaining subjects into 80% for training and 20% for validation. We used the validation data for early stopping for the neural network training. For sleep-energy, we used the training data for generating the transition probabilities and the validation data for the confusion matrix.

We evaluated sleep-energy using the test subjects. We first generate the predicted probabilities for the sleep stage at each epoch *t* and then optimise the energy of the entire sleep sequence using the simulated annealing procedure. We used the parameters *α* = 0.5, *ϵ_t_* = 0.1, and *ϵ_p_* = 0.1 unless otherwise noted. We used the accuracy, balanced accuracy and F1 Score metrics to compare the improvements obtained using sleep-energy. Finally, we used the paired Wilcoxon signed-rank test to assess statistical significance, with Holm-Bonferroni corrections for multiple comparisons. We used a significance level of 5% for the statistical tests (*p*−value < 0.05).

We trained the deep learning architecture using pytorch [34] and braindecode [35] on an Nvidia DGX with 4 A100 boards. We implemented the energy method in Python, using numpy [36], scikit-learn and scipy. The source code of the energy model and experimental results are available at https://github.com/rycamargo/sleep-energy.

## IV Results

Using sleep-energy improved the accuracy of both neural network models (U-Sleep and Chambon) on both datasets (Sleep EDFx and DRM-SUB) with high statistical significance, as shown in Table 1. The most significant improvement was 4.0% for the U-Sleep with the EDFx dataset, and the smallest was 0.7% for Chambom in the DRM-SUB dataset. The smaller Chambon model had better accuracy on the small DRM-SUB dataset, with only 20 subjects, while the larger U-Sleep was better on the EDFx dataset, with 87 subjects. The energy model also improved the balanced accuracy in all cases (Sleep-EDFx was statistically significant), which indicates that the model does not simply enhance the accuracy of the most prevalent sleep stage classes to improve the overall accuracy. Moreover, the average F1 Score was also slightly improved (although not statistically significant), which also indicates that the model maintains an equilibrium between the precision and recall of the multiple sleep stage classes.

**Figure 2:**
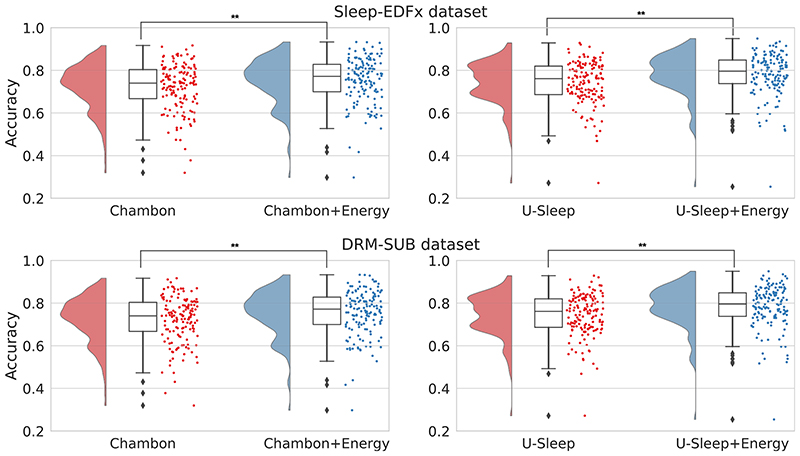
Boxplot with accuracy distribution per EEG recording before and after the energy optimisation (**p < 0.01).

Looking at the predictions for each subject (Figure 2), we see a general improvement in the accuracy when using the sleep-energy, except for outlier subjects with very low initial accuracy. In this case, the model could not improve the accuracy since it depends on the quality of the predictions from the original models to generate the confusion matrices and prediction probabilities. Also, each epoch’s neighbours must have correct predictions to adjust the system’s energy correctly.

**Figure 3:**
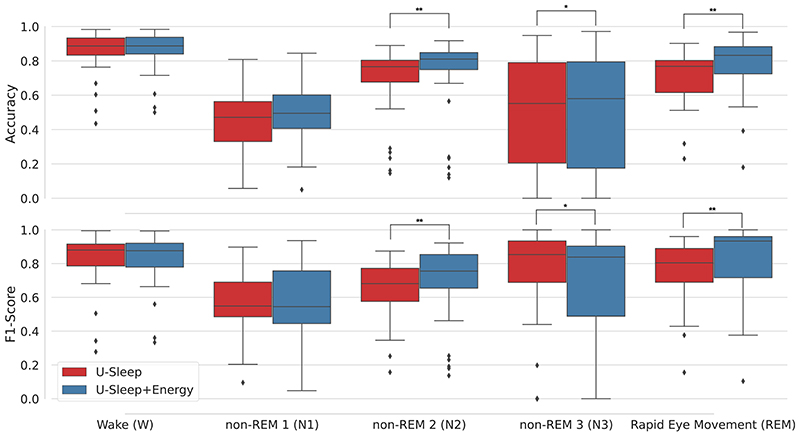
Distribution of two machine learning metrics for each sleep stage in the Sleep-EDFx dataset after optimisation of U-Sleep model predictions with sleep-energy (*p < 0.05 and **p < 0.01).

When evaluating the individual sleep stages (Figure 3), the improvements in accuracy and F1-score occurred primarily for the REM and N2 classes (*p* < 0.01). At the same time, for N3 there was a small increase in accuracy and decrease in F1-score. For W and N1 there was no statistical difference in using the energy optimisation.

**Figure 4:**
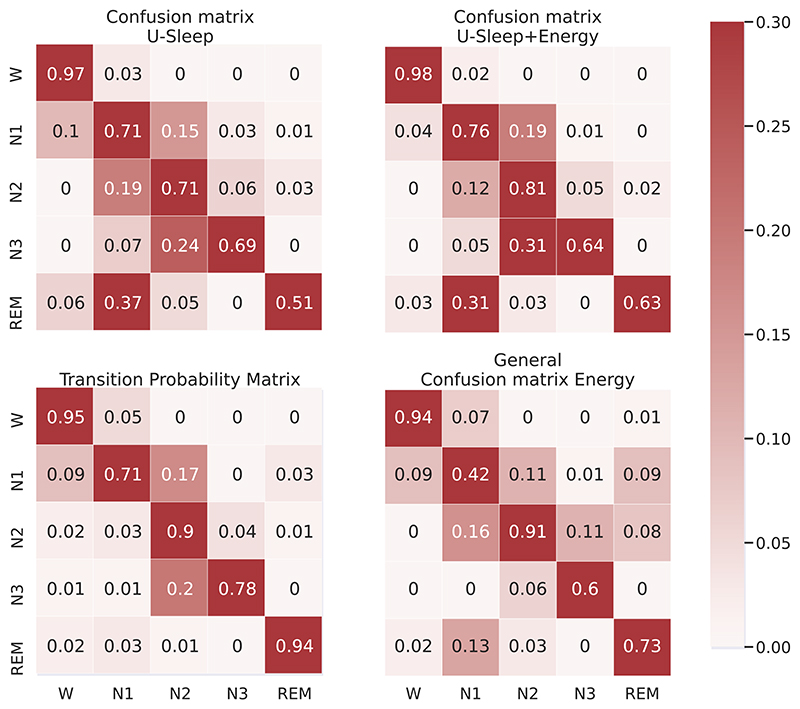
(Top): Confusion Matrix for the sleep stages predictions from the U-Sleep model for the EDFx dataset before and after sleep-energy application. Lines represent the target sleep stage, and columns the predicted stages. (Bottom): Input matrices to sleep-energy. Transition probability matrix for sleep stages on the EDFx dataset (left-hand side) and confusion matrix for the U-Sleep model using the validation set (right-hand side), where lines represent target stages and columns the predicted ones.

The improvements in the accuracy for each sleep stage class are also evident when comparing the confusion matrices for the original U-Sleep predictions and the energy-optimised ones (first row in Figure 4 for one subject). The energy method improves the prediction for most stage classes (main diagonal), except for stage N3.

**Figure 5:**
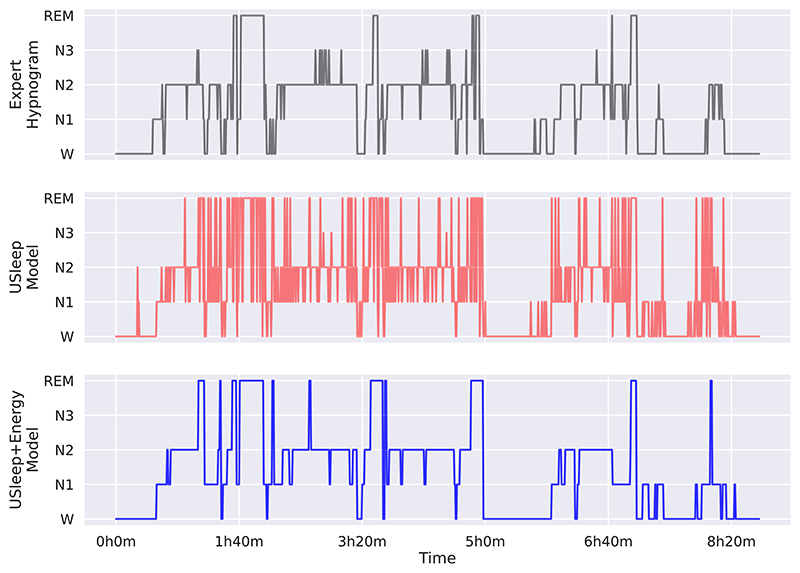
Example of a hypnogram from the EDFx dataset with the target defined by a clinical expert (top), followed by the U-Sleep predictions (middle), and the improved predictions using sleep-energy (bottom).

The origin of the accuracy improvements becomes clear when evaluating the hypnogram (Figure 5). The energy optimisation corrects the multiple mispredictions of individual epochs by adjusting them according to their neighbours and making the hypnogram less noisy and more similar to the expert hypnograms. The model depends on the initial quality of the predictions, as shown in the period before 1h40, where there were multiple predictions for REM stages. Although the sleep-energy removed most of these predictions, some remained.

The transition probability matrix used as input for sleep-energy shows that, in most cases, the sleep stage tends to stay the same in consecutive epochs (Figure 4, bottom left). There are also some transitions that never or rarely occur and cause a substantial increase in the model’s energy if maintained. But it also depends on the confusion matrix (Figure 4, bottom right) and the probability for each prediction generated by the neural network model. We should note that the confusion matrix estimated from the validation set has some differences from the test set (Figure 4), possibly due to variability among individuals. A better estimation of the confusion matrix could improve the energy method optimisations.

We also evaluate the effect of using different values for *α* in Equation 1. When *α* = 0, we are using only the prediction probability and confusion matrix information in the energy model, and with α = 1, we use only the stage transition probability matrix. We used *α* = 0.5 as the default value in all other experiments to provide a balanced contribution for the transition and prediction/confusion matrices. We can note that using *α* between 0.25 and 0.75 delivered similar results (Table 2, showing the method’s robustness to changes in this parameter value. With *α* = 1, when only information on transition probabilities is used, there was a clear decrease in all metrics, while with *α* = 0, the result is similar to that without sleep-energy.

## V Discussion

Other projects also explored using sleep stage transition probabilities to improve automatic sleep staging. For instance, Yang et al. [23] used a Hidden Markov Model (HMM) to improve the sleep stage sequences generated by a single electrode convolutional neural network model, using sleep stage transition probabilities for the HMM model. They obtained an improvement of 2.69% in accuracy and 6% in the mean F1 score in the EDFx dataset and an F1 score of 1.61% in the DRM-SUB dataset. Our energy optimisation method leveraged the information on prediction errors and probabilities from the neural networks to obtain 4.01% and 3.15% improvements in accuracy in the U-Sleep and Chambon models in the EDFx dataset. For the DRM-SUB dataset, the improvements were 2.84% and 0.68% for the U-Sleep and Chambon models. We obtained the most significant improvements when using the EDFx dataset, probably due to more data availability, as it allows a better estimation of the confusion and transition matrices.

A possible approach to use information from neighbouring is to provide the neural network classifier with the EEG signal from an epoch and its immediate neighbours. This provides a better context for the neural network to classify the epoch sleep stage. The most recent models are based on the Transformer architecture [37], [38], [39] and use the Attention mechanism to obtain context information from neighbouring epochs. It is still being determined if applying sleep-energy to the sleep stage sequences generated by these Transformer-based models could improve their results since they already use contextual information.

We did not fine-tune the model parameters, which could improve the results. For instance, we showed in Table 2 that depending on the neural network model, and dataset, different *α* values provided better results. Also, different values of of *ϵ_t_* and *ϵ_p_* in Equations 2, 3 and 4 could alter the results. These parameters could be optimised using the validation sets from the cross-validation. Nevertheless, the differences were small, as shown in Table 2 for *alpha* and reasonable values of *ϵ_t_* and *ϵ_p_*.

One possible improvement to the model is to use different sleep transition and confusion matrices, depending on the moment of the sleep period, the age and gender of the subject, and sleep abnormalities. For instance, the deep sleep N3 stage appears mostly at the beginning of sleep, while REM occurs mostly at the end. Also, time spent on deep sleep tends to decrease in older subjects, while the number of WASO (wake after sleep onset) events increases [40]. Classifying automatically if a subject has sleep abnormalities is more challenging, and multiple machine-learning techniques have been proposed [41]. Providing sleep transition and confusion matrices should generate substantial improvements to the sleep model optimisations as it will use probabilities that resemble the expected sleep stage patterns more closely.

## Figures and Tables

**Table 1:** Sleep stage prediction accuracy, balanced accuracy and average F1 score (in %) using 5-fold cross-validation (mean ± standard deviation, *p < 0.05 and **p < 0.01).

Dataset Model	Sleep-EDFx			SUB-DRM		
	Accuracy	Balanced Acc	Average F-1	Accuracy	Balanced Acc	Average F-1
U-Sleep	74.30±9.98	74.00±10.06	65.24±12.10	64.56±9.67	61.09±9.43	57.96±9.99
U-Sleep+Energy	78.31±9.94**	75.72±10.09*	68.68±12.21	67.40±10.92**	63.22±10.44	59.70±11.37
Chambon	72.16±10.91	68.94±9.76	61.28±12.06	76.78±6.69	73.36±8.76	70.89±8.39
Chambon+Energy	75.31±10.66**	70.16±10.60**	63.51±12.54	77.47±7.09**	74.14±8.96	71.54±9.07

**Table 2:** Sleep stage accuracy, balanced accuracy and average F1 score (in %) using 5-fold cross-validation for different *α* values (mean ± standard deviation).

Model	α	Sleep-EDFx			SUB-DRM		
		Accuracy	Balanced Accuracy	Average Fl Score	Accuracy	Balanced Accuracy	Average Fl Score
U-Sleep	0.00	74.76 ± 9.81	74.12 ± 9.93	65.55 ± 12.00	63.84 ± 9.36	60.35 ± 8.75	57.14 ± 9.45
	0.25	77.57 ± 9.72	75.88 ± 9.95	68.04 ± 12.19	66.03 ± 10.39	62.51 ± 9.90	59.00 ± 10.48
	0.50	78.31 ± 9.94	75.72 ± 10.09	68.68 ± 12.21	67.40 ± 10.92	63.22 ± 10.44	59.70 ± 11.37
	0.75	79.30 ± 9.46	75.12 ± 9.49	69.00 ± 11.57	67.97 ± 10.54	63.71 ± 10.30	60.41 ± 10.96
	1.00	69.59 ± 9.40	62.91 ± 8.27	57.47 ± 9.75	64.91 ± 9.51	58.43 ± 9.57	55.73 ± 10.09
Chambon	0.00	72.08 ± 10.80	68.57 ± 9.68	61.06 ± 11.96	76.70 ± 6.61	73.10 ± 8.41	70.64 ± 7.89
	0.25	74.73 ± 10.61	70.50 ± 10.33	63.41 ± 12.41	77.12 ± 6.99	73.87 ± 9.03	71.21 ± 8.93
	0.50	75.31 ± 10.66	70.16 ± 10.60	63.51 ± 12.54	77.47 ± 7.09	74.14 ± 8.96	71.54 ± 9.07
	0.75	75.63 ± 10.17	68.57 ± 10.18	62.66 ± 12.02	77.27 ± 7.43	73.81 ± 8.78	71.24 ± 9.22
	1.00	67.42 ± 10.26	59.35 ± 9.48	53.61 ± 10.52	74.99 ± 5.96	67.82 ± 7.75	66.21 ± 8.25

## Footnotes

1 As defined by the American Academy of Sleep Medicine (AASM).

2
https://www.physionet.org/content/sleep-edfx/1.0.0/

3
https://zenodo.org/record/2650142#.ZB4fs9KZOV4
